# Effects of georeferencing effort on mapping monkeypox case distributions and transmission risk

**DOI:** 10.1186/1476-072X-11-23

**Published:** 2012-06-27

**Authors:** R Ryan Lash, Darin S Carroll, Christine M Hughes, Yoshinori Nakazawa, Kevin Karem, Inger K Damon, A Townsend Peterson

**Affiliations:** 1 Rickettsial Zoonoses Branch, U.S Centers for Disease Control and Prevention, Atlanta, GA, USA; 2 Poxvirus Program, Poxvirus and Rabies Branch, U.S. Centers for Disease Control and Prevention, Atlanta, GA, USA; 3 Biodiversity Institute, University of Kansas, Lawrence, KS, USA

## Abstract

**Background:**

Maps of disease occurrences and GIS-based models of disease transmission risk are increasingly common, and both rely on georeferenced diseases data. Automated methods for georeferencing disease data have been widely studied for developed countries with rich sources of geographic referenced data. However, the transferability of these methods to countries without comparable geographic reference data, particularly when working with historical disease data, has not been as widely studied. Historically, precise geographic information about where individual cases occur has been collected and stored verbally, identifying specific locations using place names. Georeferencing historic data is challenging however, because it is difficult to find appropriate geographic reference data to match the place names to. Here, we assess the degree of care and research invested in converting textual descriptions of disease occurrence locations to numerical grid coordinates (latitude and longitude). Specifically, we develop three datasets from the same, original monkeypox disease occurrence data, with varying levels of care and effort: the first based on an automated web-service, the second improving on the first by reference to additional maps and digital gazetteers, and the third improving still more based on extensive consultation of legacy surveillance records that provided considerable additional information about each case. To illustrate the implications of these seemingly subtle improvements in data quality, we develop ecological niche models and predictive maps of monkeypox transmission risk based on each of the three occurrence data sets.

**Results:**

We found macrogeographic variations in ecological niche models depending on the type of georeferencing method used. Less-careful georeferencing identified much smaller areas as having potential for monkeypox transmission in the Sahel region, as well as around the rim of the Congo Basin. These results have implications for mapping efforts, as each higher level of georeferencing precision required considerably greater time investment.

**Conclusions:**

The importance of careful georeferencing cannot be overlooked, despite it being a time- and labor-intensive process. Investment in archival storage of primary disease-occurrence data is merited, and improved digital gazetteers are needed to support public health mapping activities, particularly in developing countries, where maps and geographic information may be sparse.

## Background

Georeferencing is an essential first step towards enabling GIS-based analyses of public health data [1,2]. It is the process by which textual descriptions of the geographic provenance of cases and diagnostic specimens are transformed into digital spatial data (longitude and latitude coordinates; “geocoding” is generally used to refer to the simpler process of adding geographic coordinates to postal addresses) [3]. The georeferencing process has been generalized into the following components: input records, reference datasets (e.g., gazetteers), and a georeferencer (the algorithm used to normalize, standardize, and match input records to the reference dataset) [4]. Ideally, the process is documented with detailed metadata [5].

The value of georeferenced public health data to state [6] or national [7,8] public health systems is clear, as it enables all spatial data analysis. However, nearly all research on the efficiency, reliability, and accuracy of georeferencing methods has relied on examples of contemporary input records and reference datasets from North America and Europe [9], possibly because georeferencing methods evolve as the availability and accuracy of reference datasets increase [4]. In contrast, our study compares three georeferencing approaches to legacy monkeypox data from villages across Central and West Africa.

Qualitative assessments of different georeferencing methods for public health data have been developed previously [10-14]. Efforts aimed at georeferencing public health data in data-poor parts of the world include trypanosomiasis in Africa [15] and malaria globally [16]. However, although these studies acknowledge the challenges faced during the georeferencing process for locations where reference data are sparse or of poor quality, they do not provide a comparison of various georeferencing methods that could guide future studies needing georeferenced disease data.

### Monkeypox background

Monkeypox (MPX) virus was first identified as an agent of human disease in 1970 in the Democratic Republic of Congo (“DRC,” then Zaire) [17]. Prior to that date, MPX virus had been isolated only from captive cynomologous monkeys [18]. MPX presents clinically in a manner nearly indistinguishable from smallpox, and thus was cause for great concern among public health officials trying to eradicate smallpox [19].

**Figure 1:**
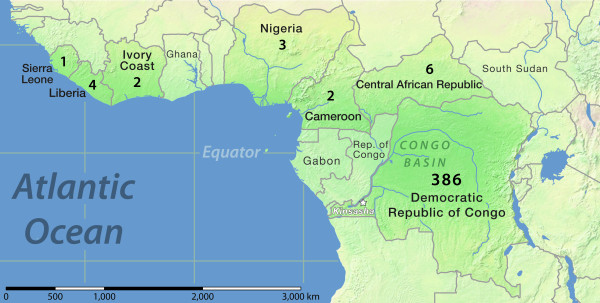
**Total reported MPX case distribution across Central and West Africa, 1970–1986**. The distribution of MPX cases in seven countries where MPX cases were reported through the joint WHO/CDC surveillance efforts, including the total number of cases identified within each county [24]. Countries labeled in gray without numbers indicate locations where additional MPX or MPX-related disease have occurred since 1986 [20-23].

During 1970–1986, human MPX cases were identified from seven countries across Central and West Africa as a result of localized active disease surveillance efforts (summarized in Figure 1). MPX cases have since been identified in Gabon [20] and the Republic of Congo [21]. Even more recently, a limited outbreak of human MPX in the United States was linked to rodents imported from Ghana [22], and human MPX cases have been identified in South Sudan [23].

An MPX-specific research agenda was outlined in 1969 to address the problems that MPX posed to the smallpox eradication campaign [25]. Under this plan, World Health Organization (WHO) Collaborating Centers in the United States and the former Soviet Union, the Centers for Disease Control (CDC), and the Moscow Research Institute for Viral Preparations, respectively, provided laboratory diagnostic services, enabling new information on MPX to be assembled. This collaborative work supported serological studies during the 1970’s and into the 1980’s [26]: surveillance activities intensified during 1981–1986 [26-28], when 21,994 specimens were tested from Congo, Ivory Coast, Sierra Leone, and Zaire [24]. During this period of intensified surveillance, 228 cases were confirmed by electron microscopy or virus culture; only 99 cases were confirmed based on serology alone, while 11 additional cases died before specimens could be collected. In all, during 1970–1986, 404 cases of human MPX disease were documented and confirmed [24].

Collection of diagnostic specimens from suspected cases of MPX followed a system established by WHO during the smallpox eradication campaign [25]. Staff at local health facilities were responsible for completing semi-standardized case forms at the time diagnostic specimens were collected from patients. Specimens and forms were sent to WHO Headquarters in Geneva, Switzerland, where they were divided and sent on to the two collaborating centers. After diagnostic testing, a diagnostic result form was generated by the lab; results were either cabled to WHO Headquarters, or sent directly to personnel in the field.

During the active surveillance period, summary information from the case forms for the 404 confirmed cases was organized in data tables. Later, WHO researchers generated a digital spreadsheet of individual case information; the geographic information in this spreadsheet enabled subsequent MPX research [29]. The spreadsheet contains five hierarchical place name fields for each case: country, region, district/zone, town, and locality. Unfortunately, details of the provenance of the data on the WHO spreadsheets are not known. In 2007, CDC researchers discovered that in the late 1980’s, after much of the initial research agenda regarding orthopoxviruses had been completed, many of the CDC laboratory diagnostic records were converted to microfilm and the originals likely destroyed. The microfilm has since been scanned digitally, and converted to PDF formats. Preliminary comparisons of data from a few case forms against the information in the WHO spreadsheet identified several inconsistencies, which served as a motivation for this study.

An active area of recent MPX ecology and epidemiology research is based on GIS mapping and modelling techniques used to search for patterns between the locations of case occurrences and geographic and environmental variables [24,29-31]. Historically, broad association of MPX virus and tropical forest was observed in early MPX research [32-34]; later, continental-scale ecological niche models showed that disease occurrence had stronger association with mean annual precipitation than with land cover [29]. Subsequent analyses at finer spatial scales constrained to within the Congo Basin, however, pointed back to proximity to dense forest [30], probably reflecting different scales and resolutions. However, studies to date have not considered the quality of the georeferencing of the case occurrence data used as model inputs—this point, although seemingly a simple methodological step, ends up being quite important.

Here, we test the hypothesis that different levels of effort invested in the georeferencing process can introduce considerable biases into geographic models of disease transmission. Specifically, we produce three georeferencing data sets for the MPX disease occurrences based on the same original WHO data, but differing in the detail and care with which they were derived. The first was based on automated georeferencing modules developed to facilitate the georeferencing process for biodiversity data (“automated data set”). Such automated approaches approximate the level of care and attention that many researchers pay to this step, and indeed exceed greatly the standards of some studies, which have depended on Internet search engines such as Google, Bing, and Yahoo maps, along with Open Street Map. The second data set, or “worked data set,” was developed by consulting a broader suite of geographic data sources to refine the first. This method explores the results one might obtain if not intimately aware of the nuances of a set of disease data. The final data set, or “researched data set,” was developed by consulting both geographic datasets and legacy CDC records (“researched data set”). This method represents the product of exhaustive searches for the greatest number of highest-quality georeferences could produce for our study system.

To compare the results of these methods, we developed ecological niche models and maps of potential MPX distributions based on each of the three occurrence data sets, and thereby can assess the effects of the different georeferencing methods on maps of MPX transmission risk (this latter defined for the purposes of this particular example as the potential for transmission at a site, given its environmental characteristics and geographic position).

## Methods

### Georeferencing

We used the point-radius approach [5] and implemented the recommended metadata architecture [35] to document the georeferencing process in the production of all three data sets. This approach captures (1) the original data, such that the lineage of information is preserved back to its source; (2) all decisions and assumptions made in the course of the georeferencing process; (3) the georeferenced coordinates, in a specified format and datum; and (4) a summary of uncertainty associated with the georeference. This summary of uncertainty represents an integration of uncertainty inherent in the geographic reference (e.g., an incomplete description), uncertainty in components of the geographic reference (e.g., “5 miles east” may be anything between 4.5 and 5.5 miles, and anything between northeast and southeast), and uncertainty in the underlying geography (e.g., the spatial footprint of the site referred to, distances among ‘multiple hits’ in matching gazetteer data). It is expressed as the radius of a circle that sums the diverse sources of uncertainty in the georeference. We relied on the MaNIS georeferencing calculator for estimating positional uncertainty [36] and excluded any locality with an uncertainty greater than 10 km.

#### Automated data set

The methods for producing the automated data sets are similar to the single-stage georeferencing methods described elsewhere [10,37]. We used the automated georeferencing facility implemented in the Biogeomancer workbench [38]. This free, web-based platform automates georeferencing by taking the WHO spreadsheet input data, and searching for matching localities in the National Geospatial-Intelligence Agency’s (NGA) GEOnet Names Service (GNS) database [39], and then automatically calculating and populating the MaNIS metadata fields [40]. We reduced the initial set of input data to unique textual locality records, and submitted the resulting table of country, state, district, municipality, and locality records to Biogeomancer for automated georeferencing.

#### Worked data set

The methods for producing the worked data sets are akin to multi-stage georeferencing methods described elsewhere [10,37], wherein we attempted to match manually input data for which satisfactory georeferences were not produced by the automated method. Here, the initial Biogeomancer output was processed further by a person knowledgeable in African geography (ATP), but without access to the case reports. Using the automated output from the Biogeomancer Workbench facility (see above) as a starter, the data were explored further, refining initial automated results using locality information on the Biogeomancer site, and incorporating additional information from additional sources: gazetteer data [41], Google Earth, and general Internet searches. The objective was to ascertain the location of each record with greater precision, and to describe uncertainty [5] more accurately. This step involved 5–30 minutes of work per locality, and the result is referred to as our “worked” dataset.

#### Researched data set

The method used for georeferencing the researched data departs considerably from the previous two methods, and may be characterized as an iterative, detailed clerical review [42]. It is distinguished from the previous two methods because it utilized legacy primary disease data to refine the input data, and it consulted a broader range of geographic reference material than those used in the automated and researched methods. The CDC legacy case form provided the basis for modifying and refining the input data, based on the assumption that the WHO spreadsheet contained transcription and other typographical errors. Additional legacy data was used to enrich the available geographic reference material, by compiling all available historic maps of MPX case locations into a common GIS map document to easily overlay and compare geographic information from different sources [17,19,24,27,32-34,43-51]. GNS geographic reference data was further supplemented with Joint Operation Graphics (JOG) topographic reference maps [52,53].

The workflow used to produce this dataset for MPX cases was iterative, as persistent and repeated searches sometimes turned up additional useful information. The initial step was to identify and resolve discrepancies between the input data from the WHO spreadsheet and the available case forms. Next, we examined all information available about individual cases to construct a sound spatial logic for identifying locations. When discrepancies were encountered, information from different sources had to be prioritized. We deemed original case forms as the most authoritative, but these records were not available for all cases. If original case forms were unavailable, the earliest published journal article reports were prioritized. If these two sources proved unhelpful, then information in review articles or marginal annotations was considered.

Once we had verified the geographic information for a given case, we began the search for a matching reference location. Our general strategy for assigning a georeference was to consult the JOG maps first, which had the finest spatial resolution, using all available information sources to find the locality on JOG maps (sometimes including preliminary GNS searches). If no location could be found or inferred there, then less-detailed data resources were used in order of decreasing precision. To expedite locating areas of interest within the JOG maps, GNS was consulted because it could be queried electronically. If a single GNS match was found, then the location could frequently be confirmed on the JOG maps and more precise coordinates recorded. If no probable match was found in GNS, or if more than one location had the same place name, then information from alternative data sources was used to guide searches. In all cases, prior to model development (see below), we discarded localities for which the uncertainty radius exceeded 10 km.

We evaluated the quality of results for each of the georeferencing methods based on completeness, positional accuracy, concordance, and repeatability [13]. Completeness is determined by the number of locations which could be matched to latitude and longitude coordinates. Positional accuracy is determined here by the spatial resolution of the geographic reference dataset. Concordance is difficult to quantify in this study, as it assesses whether the georeferenced coordinates match truthfully those referenced by the locality place name. Since this study is based on historical data for which it is impossible to revisit, our measure of concordance is the number of localities falling within the political geography boundary cited in the original data record. Repeatability is largely determined by the georeferencing methodology.

### Ecological niche model comparisons

Ecological niche modeling is a methodology that has seen extensive use in recent years [54], and that has seen increasing applications to understanding disease geography [55]. We used a simple application of the methodology, as the purpose of these analyses was only to test whether different georeferencing methodologies identify different areas as “at risk” of MPX transmission. In particular, we developed models using the Genetic Algorithm for Rule-set Prediction, or GARP [56], based on default settings, save for generating 100 random replicate models instead of 20, and derived a consensus model that summed the 10 models with lowest omission error out of the original 100 models.

We analyzed known MPX occurrences for each of the three georeferencing approaches in the context of 7 dimensions of climate drawn from the WorldClim climate data set [57]. Specifically, we used annual mean temperature, mean diurnal range, maximum temperature of warmest month, minimum temperature of coldest month, annual precipitation, and precipitation of the wettest and driest months, which represent a diverse and relatively uncorrelated environmental space in which to calibrate models [58]. All analyses were conducted at 2.5’ spatial resolution, which is equivalent to ~6.5 km near the Equator. The niche model results were summarized as maps of putative suitable conditions, and compared by means of calculation of difference maps on a pixel-by-pixel basis.

## Results

### Differences in georeferencing methods

The 404 recorded MPX cases in the WHO spreadsheet came from 231 unique localities, a figure which may vary slightly depending on whether spelling variations are interpreted as valid entries or human error. The automated method successfully georeferenced only 69/231 localities (30% match rate); the worked method successfully georeferenced 116/231 localities (50% match rate), while the researched method successfully georeferenced 106/231 localities (match rate = 46%). Match rates for each method are broken down geographically in Table 1.

**Table 1:** Comparison of georeferencing match rates across countries and sub-national units for each different method

		**Researched**		**Worked**		**Automated**	
***Country***							
**Sub-national unit**	**WHO Locations**	**Matched**	**%**	**Matched**	**%**	**Matched**	**%**
*Cameroon*	2	0/2	0	1/2	50	0/2	0
Centre	2	0/2	0	1/2	50	0/2	0
*Central African Republic*	2	0/2	0	0/2	0	0/2	0
Sangha	2	0/2	0	0/2	0	0/2	0
*Democratic Republic of the Congo*	220	99/220	45	112/220	51	67/220	30
Bandundu	37	14/37	38	23/37	62	12/37	32
**Bas Zaire**	0	0/0	n/a	0/0	n/a	1/0*	n/a
Equateur	143	62/143	43	71/143	50	38/143	27
**Haut Zaire**	3	2/3	67	9/3*	n/a	8/3*	n/a
Kasai Occidental	3	2/3	67	1/3	33	2/3	67
Kasai Oriental	31	19/31	61	6/31	19	5/31	16
Kivu	3	0/3	0	2/3	67	0/3	0
**Shaba**	0	0/0	n/a	0/0	n/a	1/0*	n/a
*Ivory Coast*	2	2/2	100	2/2	100	1/2	50
Abengourou	1	1/1	100	1/1	100	0/1	0
Haut-Sassandra	1	1/1	100	1/1	100	1/1	100
*Liberia*	2	2/2	100	0/2	0	0/2	0
Grand Gedeh	2	2/2	100	0/2	0	0/2	0
*Nigeria*	2	2/2	100	1/2	50	1/2	50
East Central	1	1/1	100	0/1	0	0/1	0
Oyo	1	1/1	100	1/1	100	1/1	100
*Sierra Leone*	1	1/1	100	0/1	0	0/1	0
Southern	1	1/1	100	0/1	0	0/1	0
**Overall**	**231**	**106/231**	**46**	**116/231**	**50**	**69/231**	**30**

The number of MPX case localities were matched at different rates in different national and sub-national units (i.e. state or province), which are expressed as fractions and percentages, relative to numbers of unique localities reported there in the WHO spreadsheet. Bolded regions in the DRC represent likely errors of commission, where more localities were georeferenced than would be expected based on the WHO spreadsheet. Asterisks identify probable specific instances of this type of error, such that calculating match rate percentages are not useful.

The georeferencing process for the researched data set is of particular interest. During this process, 48 locations were georeferenced using the input data as listed in originally in the WHO spreadsheet; georeferencing remaining localities involved careful checking against primary records and/or alternative sources of geographic information. Table 2 summarizes the relative utility of the additional data resources used: CDC legacy records and JOG maps provided the most valuable information, followed by a coarse-scale (1:1,000,000) map that provided information on 7 localities [49]; several useful articles came from Ebola virus outbreak investigations, which covered many of the same villages.

**Table 2:** Geographic information resources consulted for “researched” dataset

**Localities**	**Name**	**Reference**
43	Joint Operation Graphic’s (JOG’s)	[52]
18	Legacy CDC case forms	
	*Reports*	
4	Report of Meeting on the implementation of Post-Smallpox Eradication Policy	[49]
3	Human infections with MPX virus: Liberia and Sierra Leone	[47]
	*Articles*	
3	The role of squirrels in sustaining MPX virus transmission.	[50]
2	Ebola haemorrhagic fever in Zaire, 1976.	[59]
4	A search for Ebola virus in animals in the Democratic Republic of the Congo and Cameroon: ecologic, virologic, and serologic surveys, 1979–1980.	[46]
1	Human MPX.	[19]
1	Human poxvirus disease after smallpox eradication.	[48]
1	Four generations of probable person-to-person transmission of human MPX.	[60]
1	Results of Ebola antibody surveys in various populations groups	[61]

The number of MPX case localities which benefited from more detailed CDC legacy data and other historic materials, by resource name.

**Figure 2:**
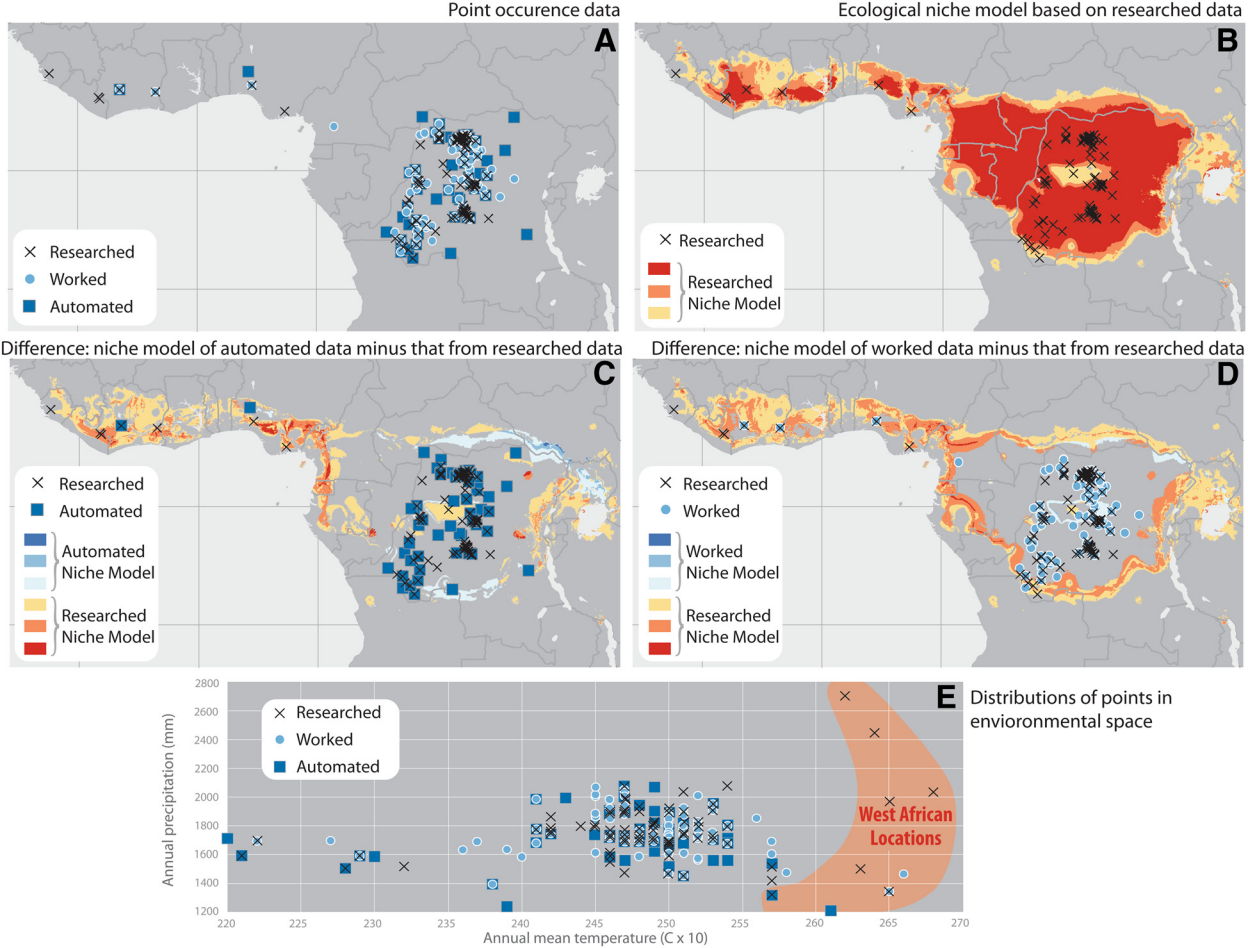
**Exploration of effects of different levels of care and detail in georeferencing of human MPX cases on derivative transmission risk maps**. Models derived from the automated and worked occurrence data differ in environmental and geographic dimensions from those based on the carefully researched occurrence data points. See text for additional detail. Red and orange areas in panels C and D are those that are more extensive in the researched data set, while blue areas are those that are less extensive. Panel E highlights portions of the ecological niche unique to the West African countries (Nigeria, Ivory Coast, Liberia, Sierra Leone) which were located using the researched method, but largely missed by the other two methods.

The above discussions of development of georeferenced public health data sets may all be inconsequential if the additional precision and documentation that they provide make no tangible difference to the outcome of analyses. That is, if the results of analyses are qualitatively the same with such high-quality data as with less-carefully-prepared data, then no reason exists to invest time in the processes outlined above. Comparing the distribution of localities of these three datasets (Figure 2A), no MPX occurrences along the eastern, southeastern, and northeastern limits of the known distribution of the pathogen were reliable, as none could be substantiated in the researched data set.

The spatial projections of the three niche models identified areas that differed consistently. In brief, the researched data set identified broader areas throughout West Africa, as well as broader areas to the southwest and east in the Congo Basin (Figure 2B). Visualizing the occurrence points in a simple environmental space (annual mean temperature X annual precipitation; Figure 2E), we see that, although researched points define most of the extremes of the distribution of the pathogen, the points with lowest annual rainfall come from the automated dataset only. Additionally, only the worked dataset includes areas of both high temperature and high precipitation.

## Discussion

The method with the best match rate overall was the worked dataset (50% match rate overall), followed by the researched dataset (46%), and finally the automated dataset (30%) (Table 1). Comparing match rates by country shows that the worked dataset achieved 100% success only in Ivory Coast, whereas the researched dataset achieved 100% success in Ivory Coast, Liberia, Nigeria, and Sierra Leone; the automated dataset did not achieve 100% success in any country. The researched data set was successful, for example, in Liberia, because a detailed map and set of site descriptions [47] were among the materials that it used. A previous study [29] georeferenced 156 of 231 locations (68% match rate), but the georeferencing methods were not documented in detail.

While comparing match rates across each country provides a metric of how well different georeferencing methods performed broadly across the continent, 220/231 (95%) of MPX cases occurred in the DRC. In the DRC, the worked method achieved a match rate of 51%, the researched method 45%, and the automated method only 30%. Issues of concordance arise, however: for example, consider numbers of cases georeferenced in the DRC regions of Bas Zaire, Haut Zaire, and Shaba. The worked method identified 9 localities in Haut Zaire, but the WHO spreadsheet indicated only three (marked with an asterisk in Table 1). The automated method had even lower concordance, identifying 8 localities in Haut Zaire, one in Bas Zaire, and one in Shaba, when the WHO spreadsheet showed three in Haut Zaire and none in the other two regions.

Additional issues of concordance may go undetected in these automated and worked datasets, as it is not entirely clear how these methods dealt with multiple ‘hits,’ i.e., several places having the same name. In the researched processing, localities were only entered into the database if the locations fell within the indicated political geographic unit, which reduced match rates by excluding some questionable localities that did have valid returns; however, it minimized the probability of including sites falsely. Under the other two methods, this conflicting evidence was clearly viewed subjectively (worked data) or managed in unknown ways depending on distances among the multiple localities (automated data).

### Information resources for georeferencing

When georeferencing historical disease data for foreign locations, this study shows that georeferencing results are improved by both supplementing geographic reference information, and consulting a variety of information sources to check and validate input data. The overall match rate improved considerably between the automated method and the worked and researched methods because the latter two methods utilized additional geographic reference information beyond a single gazetteer (e.g. GNS). While the overall match rate between the worked and researched methods were similar, the researched method used more authoritative geographic information resources. The worked method included the Falling Rain digital gazetteer [41] for which there is no metadata about its data sources or standards. In comparison, the researched method made extensive use of the JOG maps, which have very detailed standards and specifications [62].

The CDC legacy case forms were a unique and informative resource that illuminated and modified the information in the WHO spreadsheet which has previously been available to MPX specialists. These records allowed us to seek details of geographic reference in several dimensions—place of residence, location of the reporting clinic, etc. Such information may frequently not be available for other disease systems, but their utility in this study pointed clearly to the importance of tracking down all levels of documentation for disease case occurrences in such studies.

The legacy case forms posed challenges, though. They were not available for all 404 cases; four different variations of the typed form had been used; and forms were almost always completed by hand. In theory, cases for which CDC provided confirmatory testing (n = 193) should have been available; however, not all of these case forms could be located. Generally, forms captured important information, including patient identification, patient history, health facility contact information, examining physician, and regional surveillance team, and each patient was assigned a unique identification number. Specific to the geographic information on the form, a case’s place of residence was captured using a hierarchy of place names, including the following fields: name of region (e.g. administrative level-1), sub-region (e.g. administrative level-2), zone (e.g. administrative level-3), collectivité (a french term for a local government administrative unit, e.g. administrative level-4), and locality (e.g. village of residence). Only one of the four versions of the case form included the sub-region field. Two versions of the form included separate zone, collectivité, and locality fields for where the affected person was when illness began, and where the case had resided two weeks prior to onset of symptoms; however, this information was most commonly identical. One version of the form did not have separate fields for each of the hierarchical place names; rather, it asked for the “complete address” of the case, and the person completing the form filled in abbreviated field names for collectivité, zone, and region.

**Figure 3:**
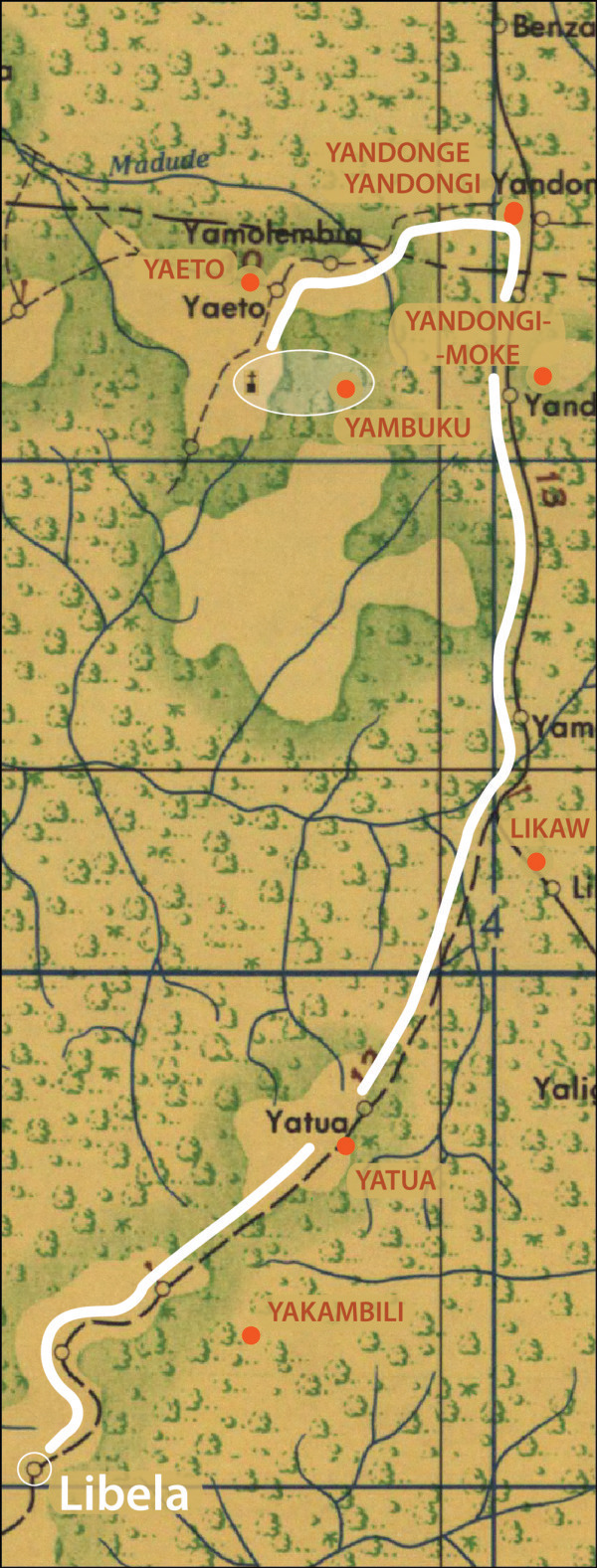
**Example of application of complex spatial logic to georeferencing a difficult locality**. A portion of a JOG map is shown, with GNS gazetteer data overlaid as orange dots with orange labels. The village of Libela did not appear on either the JOG map or in the GNS database, but anecdotal reference was made to it as being 38 km south of Yambuku [61]. Using ArcGIS, a 38 km distance (solid white line) from Yambuku Mission (church symbol on JOG map highlighted in white) to the south led to an unnamed village on the JOG map 38 km away, which could reasonably be inferred to be Libela.

The JOG maps also proved useful for overcoming the limited precision of the GNS data. It is worth noting that when localities from the GNS data are overlaid on the JOG maps in ArcGIS, the village locations between the two do not align perfectly, apparently owing to the higher spatial precision of the JOG maps (Figure 3). In GNS, nearly all Congo Basin localities have been truncated to the nearest 1’ (~2.6 km near the Equator), whereas the scale of the JOG maps provides geographic precision finer than 1 km. A limitation of both the GNS and JOG maps, is the fact that little information is known about the temporal provenance of the information in either resource. Similar temporal problems with georeferenced data have been noted elsewhere [63], and potential end users of the data must be aware that no solution is readily available.

While the GNS data set provides a helpful textual search functionality, JOG maps (which must be inspected visually by the user) allow more accurate georeferencing. Operationally, using GNS and the JOGs in tandem was the most efficient process. If a locality could be found using the text-based search in GNS, it could frequently be found and georeferenced with greater precision using the JOG maps. When a record could not be identified in GNS at the locality level, the next-higher unit place name (county, district, etc.) could frequently be found on the JOG maps, which then guided visual searches of the JOG maps for the locality—many of the place names found on JOG maps have not been captured in the GNS database. Because JOG maps were not available for our entire study area, some potential exists for spatial bias in the resulting georeferencing database. However, such areas were not omitted completely because some records could be georeferenced via other information resources, so we neglect this source of bias in our results.

The following provides an example of one of the unique and more complex instances of the georeferencing process, for the locality “Libela.” Libela was recorded as a MPX occurrence locality from a case in 1972, but was not found in either the GNS database or the JOG maps. Likely alternative spellings (e.g., Libella, Lebella, etc.) were considered, but again no matching records were found. After an Internet search using Google, a reference to Libela was identified in the proceedings of a conference on Ebola virus held in 1977, where the author notes a fatal case of possible hemorrhagic fever “in Libela (38 km south of Yambuku) [61].” Figure 3 shows a portion of a JOG map near Yambuku Mission (not labeled on the map, but noted with a church symbol, and included in the GNS database). Following the only road south from Yambuku for 38 km leads to an unlabeled populated place symbol, which we inferred to be Libela. Hence, in this example, we had to use the conjunction of GNS and JOG to identify Yambuku, and then non-standard Internet resources to find the relationship of Libela to Yambuku.

### Monkeypox transmission geography

The extra effort invested in the ‘researched’ data set impacted the results of the ecological niche models. As the data in Table 1 shows, the researched dataset matched all of the West African locations (Nigeria, Ivory Coast, Liberia, Sierra Leone), but both the automated and worked datasets failed to locate many of the cases in this region (Figure 2A). Ecological niche models generated from the results of the researched method (Figure 2B) therefore include more area in West Africa as part of their predictions. However, models generated from the results of automated (Figure 2C) and worked (Figure 2D) georeferencing methods largely do not include much of these West African locations in their predicted distribution. The ecological conditions represented by the West African locations are different than much of the rest of the MPX ecological niche, as shown in the highlighted portion of Figure 2E. Areas along the northern and southern edges of the Congo Basin were more variable in the effects of researching data points, as the signals from the worked and automated data sets differed for these areas.

Even without the modeling step, the exercise of investigating each occurrence record in great depth was illuminating, and the linking of individual diagnostic results with each unique location proved insightful. No researched data point fell in the eastern quarter of the Congo Basin. Biologically more importantly, however, no researched data point comes from the Republic of the Congo, on the west side of the Congo River above Kinshasa. This latter area has not seen massive political conflicts, so this absence may in fact be real; research is underway into the causes of this lack of records from the region. Since the relational database created was able to incorporate data on confirmatory lab test as well, we can state that laboratory confirmation of MPX by viral culture occurred in 70 (66%) of the 106 localities in the researched data set, a higher standard for disease confirmation than serology testing alone. Hence, earlier studies based on the less carefully researched WHO spreadsheet [29] must be taken with a grain of salt: quite simply, different georeferencing have very-real implications for results of mapping exercises.

## Conclusions

This paper contributes uniquely because we document the difficulties and limitations in the available methods for georeferencing under challenging conditions, namely historic disease data in foreign locations with poor geographic reference information. We demonstrate the utility of institutional legacy data and importance of consulting a variety of geographic data resources to the process of georeferencing. We show meaningful differences in the resulting MPX distribution depending on the georefrencing method chosen. While other studies have encountered and identified similar difficulties to georeferencing historic public health data from developing countries [15,16], the MPX data used in this study are even older; we believe that our results may help other researchers in the future to plan strategically for georeferencing other historic public health data sets. Elsewhere, analyses are appearing in the literature using ecological niche modeling or other related GIS based modeling methods to examine disease distributions in various locations and at various spatial scales e.g., [64-67]. Too often, however, occurrence data are used without careful introspection or the georeferencing process is executed without detailed attention.

Such concerns have seen considerable discussion and development in the biodiversity informatics world [5,68-70]. In public health, a clear and robust argument of the need for georeferenced health data was put forth nearly 15 years ago [71]. Since then, a large amount of research has focused on georeferencing domestic disease occurrences [1,11,72-74]. The work herein, like that of Serebriakova [75], suggests that greater investment in georeferencing resources for international public health research is needed, and that legacy map library collections should be used to fill gaps in digital gazetteer data [76]. In this vein, automated approaches to extracting information from scanned maps [77] may offer even greater efficiency than manual digitizing. Discussions have begun as regards alternative formats for capture of human disease occurrence data [78,79], but much more contemplation is needed, owing to differences in disease surveillance systems and geographic information infrastructure around the world. Emerging technologies may be one way of strengthening public health surveillance capacity, such as monitoring Twitter feeds [80], and other types of mobile communications [81]. In light of the ongoing threat posed by emerging and re-emerging infectious diseases [82], it seems most advantageous to initiate a focus on constructing high-quality, well-documented geographic summaries of primary disease data.

## Competing interests

The authors claim no competing interests.

## Authors’ contributions

RRL, DSC, and ATP designed and executed this study, and drafted the manuscript. CMH provided access to existing CDC Poxvirus Program data, and assisted in interpreting legacy data. YN provided extensive comments on the manuscript. KK and IKD supported design and execution of study, and assisted in accessing and interpreting legacy data, while also providing constructive comments on the manuscript. All authors read and approved the final manuscript.

## Authors’ information

Ryan Lash was a Guest Researcher in the Poxvirus and Rabies Branch during the time this research took place. At the time of writing, Ryan Lash’s affiliation at CDC is as an Oak Ridge Institute for Science and Education (ORISE) Fellow in the Rickettsial Zoonoses Branch, while he pursues a PhD in the Geography Department, University of Georgia, Athens, GA.
